# APOE3-Christchurch variant enhances neurovascular support functions of iPSC-derived mesenchymal stromal cells

**DOI:** 10.3389/fmolb.2026.1778856

**Published:** 2026-06-01

**Authors:** Paula J. Rodriguez Martinez, Aria R. Yslas, Yasuteru Inoue, Tammee M. Parsons, Samantha K. Baker, Wenyan Lu, Ana-Caroline Raulin, Takahisa Kanekiyo

**Affiliations:** 1 Department of Neuroscience, Mayo Clinic, Jacksonville, FL, United States; 2 Mayo Clinic Center for Regenerative Biotherapeutics, Jacksonville, FL, United States

**Keywords:** angiogenesis, APOE3 R136S, BBB, iPSCs, metabolic activity, MSCs, neural outgrowth

## Abstract

Aging and neurodegenerative disorders like Alzheimer’s Disease (AD) are associated with progressive dysfunction of the blood-brain barrier (BBB) and neurovascular unit (NVU), contributing to impaired vascular integrity and neuronal vulnerability. Apolipoprotein E (APOE) is a key regulator of neurovascular function, and the rare *APOE3-R136S* “Christchurch” variant (*APOE3Ch*) confers protection against AD. Mesenchymal stromal cells (MSCs) represent a promising cell-based therapy for the treatment of neurodegenerative diseases due to their paracrine effect exerted on vascular and neural cells. To investigate how *APOE3Ch* influences MSC-mediated neurovascular support, we used isogenic iPSC-derived MSCs (iMSCs) with homozygous *APOE3Ch* or *APOE3*. We found that *APOE3Ch* iMSCs have stronger immunosuppressive effect on LPS-induced NFκB activation of THP1 cells. *APOE3Ch* iMSCs also enhanced endothelial barrier resistance and angiogenic capacity compared to *APOE3* iMSCs when directly co-cultured with endothelial cells. In addition, conditioned medium from *APOE3Ch* iMSCs promoted neurite outgrowth more efficiently than that from *APOE3* iMSCs. Metabolic profiling revealed differences between *APOE3Ch* and *APOE3* iMSCs, suggesting altered metabolic resilience. Together, these findings demonstrate that iMSCs support vascular and neuronal function through paracrine mechanisms and suggest that *APOE3Ch* variant improves specific aspects of MSC-mediated neurovascular support. This work highlights the potential of combining MSC-based therapies with protective *APOE* variants to target BBB and NVU dysfunction in aging and neurodegeneration.

## 1 Introduction

Older age is the main risk factor for several types of dementia, including Alzheimer’s Disease (AD). AD is a progressive neurodegenerative disorder accounting for 60%–80% of dementia cases. It presents at the clinical level with memory loss and cognitive decline that exceed those seen in healthy aging. Pathologically, AD is characterized by two distinct hallmarks: extracellular amyloid-beta (Aβ) plaques and intracellular tau neurofibrillary tangles (NTFs), both of which eventually lead to neuroinflammation, synaptic dysfunction, and widespread neuronal death (Alzheimer’s Association, 2022). Both amyloid and tau pathologies have been reported to further compromise the cerebrovascular system including blood-brain barrier (BBB) integrity, thus perpetuating chronic inflammation and neurodegeneration (Chen et al., 2025). The BBB is a highly selective, semi-permeable membrane that separates the central nervous system from the rest of the body, regulating the movement of molecules between the two compartments and preventing leakage (Banks et al., 2021; Wu et al., 2023). The BBB is specifically located on the capillary bed and is composed of brain endothelial cells (BECs) (Banks et al., 2021). They line up the lumen of blood vessels, forming tight junctions that allow for the passage of molecules from the blood to the brain (Ludewig et al., 2019). BECs, together with neurons, glial cells, and pericytes, form the neurovascular unit (NVU) (Procter et al., 2021; Attwell et al., 2016).

*APOE* ε4 is the strongest genetic risk factor for AD. The ε4 allele of *APOE* is one of three major alleles, with ε2 being a protective variant and ε3 considered neutral. APOE in a 299-amino acid long glycoprotein which primary function is to transport lipids. In the brain, it is largely secreted by astrocytes; other cell types, including pericytes, have been reported to contribute to APOE production albeit at lower levels. The major APOE isoforms, APOE2, APOE3, and APOE4 are structurally distinct with Cys to Arg substitutions occurring at positions 112 and 158 (APOE2: Cys112/Cys158; APOE3: Cys112/Arg158; APOE4: Arg112/Arg158), which modulates their respective function (Raulin et al., 2022). In addition to clear links between the presence of APOE4 and worsening amyloid and tau pathologies, APOE4 has also been linked to NVU dysfunction compared to APOE3 in both post-mortem human tissue and mouse models of AD (Yamazaki et al., 2019). APOE4 negatively impacts BBB structure and function (Nishitsuji et al., 2011; Jackson et al., 2022). Of note, the rare *APOE3-R136S* “Christchurch” (*APOE3Ch*) mutation corresponding to an Arg to Ser mutation at position 136 on an APOE3 background (Wardell et al., 1987) was linked to resistance to AD when homozygosity for *APOE3Ch* led to a remarkable delay in onset of familial AD driven by an autosomal-dominant *PSEN1* E280A mutation (Arboleda-Velasquez et al., 2019). Although how the Christchurch mutation affects different aspects of AD, including amyloid and tau pathologies has been increasingly investigated (Liu et al., 2024), the vascular consequences of APOE3Ch remain largely unexplored.

Mesenchymal Stromal Cells (MSCs) are multipotent stem cells that have the capability of differentiating into cells of a mesodermal lineage, such as adipose tissue and bone (Keating, 2006). In the brain, they are predominantly located in perivascular compartments, and they share phenotypic similarities with pericytes. MSCs secret a broad range of trophic, immunomodulatory, and vascular-stabilizing factors, rendering them prime candidates for BBB repair, immunomodulation, and neuronal support. Induced pluripotent stem-cell (iPSC)-derived MSCs (iMSCs) recapitulate the core MSC function (Buitrago et al., 2024), while offering a scalable and reproducible source suitable for therapeutic applications (Gong et al., 2025). Thus, we investigated the therapeutic potential of iMSCs coupled with the protective properties of *APOE3Ch* on neurovascular function, including barrier integrity, angiogenesis, metabolism, and neuronal support.

## 2 Materials and methods

### 2.1 Generation and maintenance of iPSC-derived MSCs

Human isogenic induced pluripotent stem cell (iPSC) lines with homozygous *APOE3* and *APOE3Ch* derived from KOLF2.1J (Pantazis et al., 2022) were used for the generation of iMSCs. iPSCs plated on Matrigel-coated (Corning) dishes were maintained in complete mTeSR1 Plus medium (STEMCELL Technologies). Upon reaching 80% confluency, iPSC colonies were dissociated into single cells using Accutase (STEMCELL Technologies). Cells were plated in a round-bottom 96-well plate (Thermo Scientific) at a density of 45,000 cells/cm^2^ in iMSC-induction medium consisting of IMDM (Gibco) supplemented with 20% KnockOut SR (Gibco), 0.1 mM non-essential AA (Gibco) and 0.1 mM 2-mercaptoethanol (Sigma-Aldrich) to allow the formation of embryoid bodies (EBs). 10 μM Y-27632 (ROCK inhibitor) was added to aid with cell survival post-passaging. After 48 h, 10–12 EBs per well were transferred to a non-adherent 6-well plate (STEMCELL Technologies) using wide-bore pipette tips and cultured for an additional 3 days in fresh iMSC-induction media. EBs were then transferred to a 1% gelatin-coated (Sigma-Aldrich) 6-well plate with fresh iMSC-induction media, slightly broken up by pipetting for better adherence. Non-attached cells were removed after 3 days, and the media was substituted for iMSC-induction medium supplemented 10 ng/mL TNFβ1 (R&D Systems, USA) to facilitate transition to MSC-like cells. Media was replaced after 2 days with iMSC-maintenance media consisting of α-MEM medium (Gibco) supplemented with 5% Fetal Bovine Serum (FBS, Thermo Scientific) and 1X Glutamax (Gibco). Media changes were performed biweekly until cells reached 90% confluency. When ready to passage, EBs were discarded, and iMSCs were dissociated using TrypLE Express (Gibco). Cells were re-plated in a 1%-gelatin coated dish at a density of 20,000 cells/cm2 in iMSC-maintenance media. These conditions were retained until iMSCs reached passage 3. All cell cultures were maintained at 37 °C in 5% CO_2_ conditions.

### 2.2 Fluorescence-activated cell sorting (FACS)

Passage three iMSCs were phenotypically characterized by presence of MSC surface markers CD73, CD90, and CD105 using standard flow cytometric analysis. Cells were harvested, washed with PBS and stained with anti-CD73, -CD90 and -CD105 antibodies from the MSC Characterization Panel (STEMCELL Technologies). A negative antibody cocktail was used for gating (Supplementary Figure S1A). FACS were conducted using Human MSC Analysis Kit (BD Stemfow™, 562,245) and Data were analyzed using an Attune NxT Flow Cytometer and analyzed using FlowJo™ Software (BD Biosciences). Unstained iMSC were used as a negative control for staining (Supplementary Figure S1B, C). iPSC and THP1 cells were used to validate the specificity of antibodies (Supplementary Figure S1D, E), with iPSCs negative for CD73 and CD105 and THP1 cells negative for CD90 (Moslem et al., 2015; Holken and Teusch, 2023).

### 2.3 THP1 NF-κB luciferase

Functional validation was performed by assessing immunomodulation properties of iMSCs. THP1 NF-κB-Luc2 cells (THP1, ATCC, UK, Cat No. TIB-202-NFkB-LUC2) were thawed in complete THP1 media consisting of RPMI-1640 medium (Gibco) supplemented with 10% FBS (Thermo Scientific), 1 μg/mL puromycin (Fisher Scientific) and 0.05 mM 2-mercaptoethanol (Sigma-Aldrich) and cultured in suspension for 2 days. Cells were centrifuged 125 *g* for 8 min and activated with LPS (Sigma-Aldrich) at 100 ng/mL in complete THP1 media. After a 6 hour-activation period, cells were centrifuged, washed, and resuspended in complete THP1 media. Cells were then plated on a 6-well plate at a density of 500,000 cells/mL. iMSCs were plated on 6-well 0.4 µm pore Thincerts (Greiner Bio-One) at a density of 11,000 cells/cm^2^ and incubated overnight. iMSC-containing Thincerts were then discarded and THP1 cells were collected, centrifuged, and resuspended in 1 mL of complete THP1 media for counting. 50,000 THP1 cells in 100 µL of complete THP1 media were then plated in white 96-well plates (Thermo Scientific). Luciferase activity was evaluated using the ONE-Glo Luciferase Reagent (Promega) following manufacturing instructions. Luminescence was read using a Synergy™ H1 microplate reader (BioTek Instruments).

### 2.4 Reverse transcriptase-quantitative polymerase chain reaction (RT-qPCR)

*APOE* gene expression levels in iMSCs were evaluated by RT-qPCR. Total RNA was isolated using the Macherey-Nagel NucleoSpin RNA Plus kit (Fisher Scientific), followed by reverse transcription with the iScript cDNA synthesis kit (Bio-Rad). qPCR was performed with SYBR Green Supermix (Bio-Rad) and run in triplicates using the QuantStudio 7 Flex Real-Time PCR System (Fisher Scientific). The following primers were used: *APOE*, forward: 5′-CGTTGCTGGTCACATTCCT-3′, reverse: 5′- CTCAGTTCCTGGGTGACCTG-3′, *GAPDH*, forward: 5′-GTCTCCTCTGACTTCAACAGCG-3′, reverse: 5′- ACCACCCTGTTGCTGTAGCCAA-3’. Relative gene expression of *APOE* was calculated using the 2^−ΔΔ(CT)^ method, with *GAPDH* as the housekeeping gene.

### 2.5 Western blot

Intracellular APOE levels in iMSCs as well as secreted APOE were quantified by Western blot. iMSC culture media was collected and cells were lysed using RIPA buffer supplemented with protease (cOmplete, Roche) and phosphatase inhibitors (PhosphoStop, Roche). RIPA lysate and cultured media samples were prepared in 4x Laemmli Sample Buffer (Bio-Rad) supplemented with 10% 2-mercaptoethanol and boiled at 95 °C for 5 min. Proteins were resolved by Sodium Dodecyl Sulfate–PolyAcrylamide Gel Electrophoresis (SDS-PAGE) on a 4%–20% precast PROTEAN TGX gel (Bio-Rad) in 1X Running Buffer (Bio-Rad), at 120V for 80 min. Proteins were then transferred onto an Immobilon-P PVDF membrane (Sigma Aldrich) in 1X Transfer Buffer (Bio-Rad) supplemented with 20% methanol at 230 mA for 3 h. Membranes were blocked in blocking buffer consisting of 5% non-fat milk in phosphate-buffered saline (PBS) for 45 min, washed with PBS-T (PBS, 0.05% Tween-20) and incubated with primary antibody hAPOE (K74180B, Meridian Life Sciences; 1:1,000 in blocking buffer) overnight at 4 °C on a shaker. Membranes were then washed with PBS-T and incubated with secondary antibody (sc-2354, Santa Cruz; 1:2,000 in blocking buffer) for 1 h at room temperature. Membranes were developed with SuperSensitive ECL (Fisher Scientific) for 5 min and imaged using the Chemidoc Imaging System (Bio-Rad). Bands were then quantified using ImageJ/Fiji.

### 2.6 Trans-endothelial electrical resistance (TEER) in endothelial cells co-cultured with iMSC

Immortalized human Brain Microvascular Endothelial Cells (hBMECs, ScienceCell, Cat No. 1000) were thawed in fibronectin-coated (ScienceCell) flasks in EGM-2 MV Microvascular Endothelial Cell Growth Medium (EGM-2 MVM ECGM, Lonza). Biweekly media changes were performed until cells reached 90% confluency. 24-well 0.4 µm-pore Thincerts were placed upside down on a 20 cm cell-culture treated dish (Greiner Bio-One). iMSCs were passaged using TrypLE Express and plated on the underside of the Thincerts at a density of 21,000 cells/cm^2^ and left to settle in the incubator overnight. Thincerts were flipped and placed in a 24-well plate (Greiner Bio-One) with regular iMSC-maintenance media. hBMECs were detached using Trypsin EDTA (Gibco) and plated on the opposite face of the Thincert at a density of 16,000 cells/cm^2^ in EGM-2 MVM ECGM. Trans-Endothelial Electrical Resistance (TEER) was measured daily for 7 days using an EVOM3 instrument (Precision Instruments) and a STX2 electrode (Precision Instruments). Daily medium changes of EGM-2 MVM ECGM and iMSC-maintenance media were also performed after each TEER reading.

### 2.7 Immunostaining and imaging of thincerts

Following the 7-day co-culture of hBMECs and iMSCs, both sides of the trans-well inserts were fixed with methanol for 10 min, washed 3 times with PBS and permeabilized with 0.3% Triton X-100 (Sigma-Aldrich) for 30 min. Inserts were blocked with Protein Block Serum-Free Ready-To-Use (Agilent Technologies) solution for 1 h. Primary antibody incubation was done using Background-Reducing Dilution Buffer (Agilent Technologies) overnight at 4 °C on a shaker. The primary antibodies used are as follows: ZO-1 (mouse monoclonal IgG, 1:200, Cat. No. 33–9100, Invitrogen) VE-Cad (mouse monoclonal IgG, 1:50, Cat. No. MAB9381, R&D Systems), Claudin-5 (mouse monoclonal IgG, 1:200, Cat. No. 35–2500, Invitrogen), Occludin (rabbit polyclonal IgG, 1:400, Cat. No. 27260-1-AP, ProteinTech) PDGRF-β (rabbit polyclonal IgG, 1:200, Cat. No. sc-339, Santa Cruz Biotechnology). Afterwards, samples were washed 3 times with PBS and then secondary antibodies anti-mouse IgG (AlexaFluor 568, 1:500, Cat. No. A10037, Invitrogen) and anti-rabbit IgG (AlexaFluor 488, 1:500, Cat. No. A11008, Invitrogen) were added. Samples were further incubated for 2 h at room temperature in the dark on a shaker. After washing 3 times with PBS, Thincert membranes were cut out and mounted on glass slides for imaging. Confocal images were acquired using a ZEISS LSM510 microscope with a ×20 objective lens with ×20482028 pixels per slice and 19 slices per image. Z-stacks were projected into a single image using ImageJ/Fiji maximum intensity projection.

### 2.8 Endothelial tube formation and imaging

Angiogenic potential of iMSCs was investigated by their impact on endothelial tube formation through direct co-culture, or from secreted factors in conditioned media. Immortalized human Cerebral Microvascular Endothelial Cells (hCMEC/D3, SCC066, MilliporeSigma) were thawed in rat tail collagen-coated (MilliporeSigma) flasks in EGM-2 MVM ECGM (ECGM). Biweekly media changes were performed until cells reached 90% confluency and were ready to passage. 50 uL of Growth Factor Reduced Matrigel (Corning) was added to a glass-bottom 96-well plate (Corning), making sure no bubbles were present, and kept in an incubator for 30 min hCMEC/D3s were detached using Trypsin EDTA (Gibco), plated at a density of 18,750 cells/cm^2^ in ECGM and left to settle for 30 min. For co-culture conditions, iMSCs were detached with TrypLE Express and plated on top of the hCMEC/D3s at a density of 6,250 cells/cm^2^ in serum-free iMSC media (SF-MSCM) consisting of α-MEM medium and 1X Glutamax, the final medium being 1:1 EGCM:SF-MSCM. For conditioned media conditions, the media of iMSC-confluent dishes was changed to SF-MSCM and cells were conditioned for 3 days. Conditioning did not lead so any significant cell death (<5% on average). Prior to the start of the experiment, conditioned media (CM) was centrifuged at 300 *g* for 3 min and added to the hCMEC/D3, achieving a 1:1 EGCM:CM medium composition. Both experimental conditions were incubated at 37 °C in 5% CO_2_ for 6 h. Following incubation, samples were washed once with HBSS (Gibco) prior to addition of the Calcein AM live stain (Invitrogen) at 2 µM in HBSS. Samples were incubated in the dark at room temperature for 30 min and washed once with HBSS before imaging. Confocal images were then acquired using the ZEISS LSM510 microscope. Mesh count, corresponding to the number of closed polygonal areas formed by interconnected endothelial tubes, was determined manually. Outliers were removed using the interquartile range rule, resulting in the exclusion of a single data point in the SF-MSCM, no iMSC group (Supplementary Figure S2B).

### 2.9 Generation of iPSC-derived neurons and neurite extension assay

iPSC colonies were dissociated into a single-cell suspension using Accutase and plated in STEMdiff Neural Induction Medium (NIM, STEMCELL Technologies) supplemented with 10 µM ROCK inhibitor on Aggrewell 800 plates (STEMCELL Technologies) pre-treated with Anti-Adherence Rinsing Solution (STEMCELL Technologies) at a density of 525,000–1,575,000 cells/cm^2^ and centrifuged at 100 *g* for 3 min. Half-media changes were performed daily for the following 4 days to allow for EBs formation. EBs were then transferred into a Matrigel-coated 6-well plate with fresh NIM. Daily medium changes with NIM were performed for the following 6 days to allow the formation of neural rosettes. Neural rosettes were visually assessed using an EVOS M5000 digital microscope (Invitrogen), manually selected and plated onto a new Matrigel-coated 6-well plate with fresh NIM for 6 days with daily NIM full-media changes. Rosettes were then dissociated using Accutase and replated on Matrigel-coated 10 cm dishes with STEMdiff Neural Progenitor Basal Medium (NPM, STEMCELL Technologies) supplemented with 10 µM ROCK inhibitor for neural progenitor cell (NPC) specification. Cells were cultured for an additional 7–14 days with daily NPM media changes. Cells were passaged a minimum of 3 times prior to neuronal differentiation.

NPCs for differentiation into iPSC-derived neurons (iN) were dissociated using Accutase and plated at a density of 3,300 cells/cm^2^ in BrainPhys (STEMCELL Technologies) with 10 µM ROCK inhibitor onto a glass-bottom 96-well plate coated with 15 μg/mL poly-L-ornithine (PLO, Fisher Scientific) in PBS and 5 μg/mL laminin (STEMCELL Technologies) in DMEM/F-12. Half-media changes were performed every 3–4 days for a week. iMSC conditioned media was prepared by culturing confluent iMSCs in BrainPhys for 3 days prior to the start of the experiment. Conditioning with BrainPhys did not lead so any significant cell death (<5% on average). Moreover, iMSCs retained their mesenchymal identity post BrainPhys exposure as shown by FACS (Supplementary Figure S3). Conditioned media was collected and centrifuged at 300 *g* for 3 min. BrainPhys was carefully removed and replaced with iMSC conditioned media. Biweekly half-media changes were performed with the iMSC conditioned media for the following 2 weeks.

iN were fixed with 4% PFA (Thermo Scientific) for 15 min, washed with PBS and permeabilized with 0.3% Triton X-100 in PBS for 30 min. Cells were blocked in cell blocking buffer (PBS with 1% Bovine Serum Albumin (BSA, Miltenyi Biotec) and 0.01% Triton X-100) for 1 h iN were incubated with TUJ1 primary antibody (mouse monoclonal IgG2a, 1:300, ab78078, Abcam) diluted in cell blocking buffer overnight at 4 °C on a shaker. Cells were washed with PBS and incubated with anti-mouse IgG (AlexaFluor 647, 1:500, Cat. A21235, Invitrogen) and DAPI for 2 h at room temperature in the dark on a shaker. Fluorescent microscopy images were obtained with a KEYENCE BZ-X810 microscope. Fluorescent microscopy images were analyzed using the ImageJ/Fiji plugin Simple Neurite Tracer (SNT) as described by Ferreira et al., 2014.

### 2.10 Mitochondrial stress test

iMSCs were plated on a Seahorse XF96 microplate (Agilent Technologies) at a density of 375,000 cells/cm^2^ overnight in iMSC-maintenance media. The Seahorse XF Mito Stress Test (103,015–100, Agilent Technologies) was performed following the manufacturer’s protocol. Briefly, cells were equilibrated in XF Phenol-red free DMEM medium containing 10 mM glucose, 1 mM pyruvate, and 2 mM glutamine (Agilent Technologies) for 1 h prior to experimentation. Oxygen consumption rate (OCR) was measured in a Seahorse XFe96 analyzer (Agilent Technologies), following sequential injections of 1.5 µM oligomycin, 0.5 µM FCCP and 0.5 µM Rotenone/Antimycin per well. iMSCs were fixed with 4% paraformaldehyde (PFA) immediately after recording and stained with DAPI. OCR data was normalized to *APOE3* iMSC cell number and related measures were determined using the associated Wave software.

### 2.11 Data analysis

All quantitative data was analyzed using Microsoft Excel and GraphPad Prism 10.4.1, and all microscopy images were analyzed using ImageJ/Fiji. For statistical analyses, p > 0.05 was regarded as non-significant, with specific statistical tests indicated in the figure legends.

## 3 Results

### 3.1 *APOE3Ch* iMSCs exhibit typical MSC identity

**FIGURE 1:**
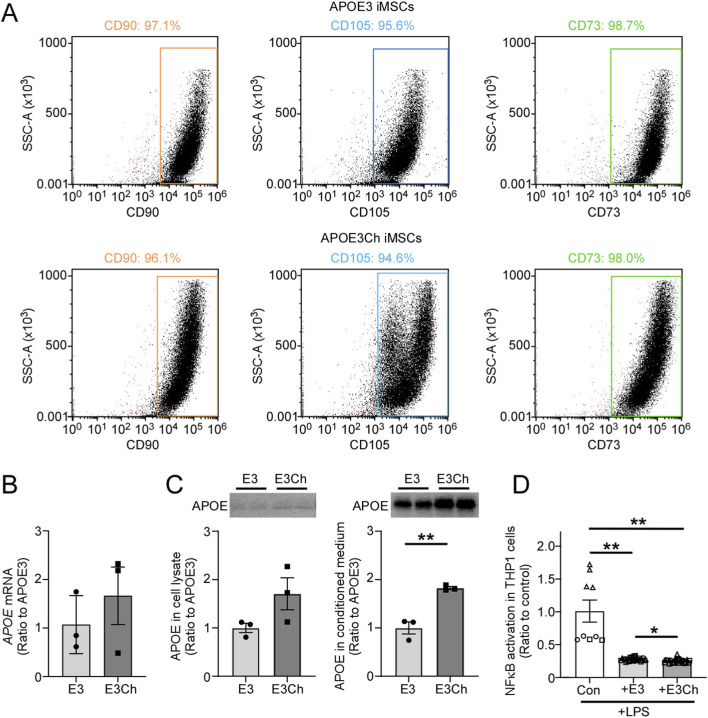
Generation and validation of iPSC-derived MSCs with *APOE3* or *APOE3Ch*. **(A)** Fluorescence-Activated Cell Sorting (FACS) analysis confirming MSC identity showing positive markers CD73, CD90 and CD105 expressed in >95% of the cell population. **(B)** Quantification of *APOE* mRNA level by RT-qPCR, normalized to housekeeping gene *GAPDH* and expressed as fold change from the *APOE3* iMSC group. Data shown as mean ± SEM (n = 3 independent differentiation batches per line). p > 0.05 by two-tailed unpaired Student’s t-test. **(C)** Densitometric of Western blot quantifying APOE levels in iMSC cell lysate and conditioned medium, expressed as fold change from the *APOE3* iMSC group. Data shown as mean ± SEM (n = 3 independent differentiation batches per line). **p < 0.01 by two-tailed unpaired Student’s t-test. **(D)** Quantitative analysis of LPS-induced NF-kB reporter activity measured by luminescence in activated THP1 cells cultured alone (Con) or indirectly co-cultured with either *APOE3* (+E3) or *APOE3Ch* (+E3Ch) iMSCs. Data was normalized to the +E3 condition and represented as mean ± SEM (n = 3 independent experiments, technical replicates from each experiment are represented by a distinct symbol, for display only). **p < 0.01, *p < 0.05 by mixed-effects model on n = 3 independent experiments with the Geisser-Greenhouse correction to account for matched values, and Tukey’s *post hoc* multiple comparisons test.

To uncover the comparative effects of *APOE3Ch* in MSCs, isogenic *APOE3* and *APOE3Ch* iPSC lines were differentiated into iMSCs. Mesenchymal identity of both lines was verified by FACS through expression of MSC surface markers CD73, CD90 and CD105 (Figure 1A; Supplementary Figure S1). RT-qPCR analysis did not detect significant differences in *APOE* mRNA levels between *APOE3* and *APOE3Ch* iMSCs (Figure 1B). In contrast, *APOE3Ch* iMSCs had significantly higher APOE protein levels in media than *APOE3* iMSCs while there were no significant differences in cell lysates as assessed by densitometry analysis of western blots (Figure 1C). The iMSCs were then functionally characterized by assessment of their respective immunomodulatory effects. LPS-activated THP1 NF-κB-Luc2 reporter cells were co-cultured either with, or without, iMSCs and activation of the NF-κB cell signaling pathway that regulates inflammation was indirectly quantified by measuring luciferase activity. The presence of iMSCs significantly reduced the activation of the NF-κB pathway in THP1 cells compared to THP1s activated in the absence of iMSCs, where *APOE3Ch* iMSCs showed slightly stronger effect than *APOE3* iMSCs (Figure 1D).

### 3.2 *APOE3Ch* iMSCs enhance endothelial barrier integrity

**FIGURE 2:**
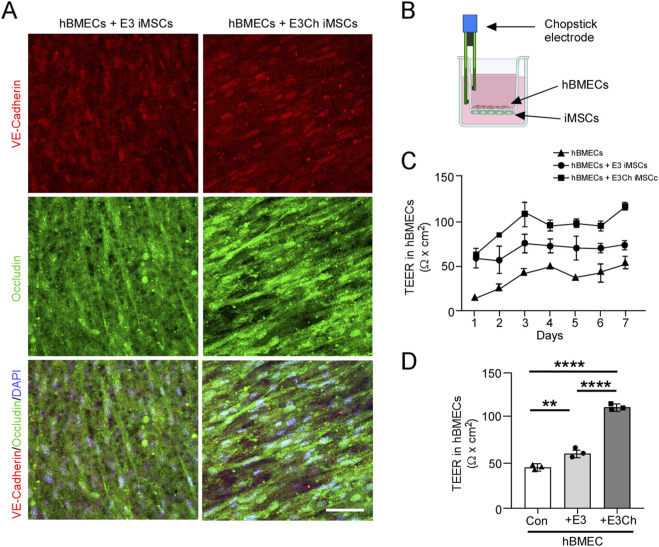
*APOE3Ch* iMSCs improve endothelial barrier integrity. **(A)** hBMECs stained for VE-Cadherin, Occludin, and DAPI after co-cultured with *APOE3* iMSCs or *APOE3Ch* iMSCs. Scale bar = 50 µm. **(B)** Schematic of TEER measurement assay setup created with BioRender.com. **(C)** Graph showing blank-corrected quantitative TEER values measured daily over the course of 7 days for hBMECs co-cultured with or without *APOE3* iMSCs or *APOE3Ch* iMSCs. Data expressed as mean ± SEM (n = 3 independent experiments). **(D)** Comparison of TEER values on day 7. Data represented as mean ± SEM (n = 3 independent experiments). **, p < 0.01, ****p < 0.0001 by One-way ANOVA with Tukey’s *post hoc* multiple comparisons test.

To investigate the effects on endothelial integrity, hBMECs were co-cultured with either *APOE3* or *APOE3Ch* iMSCs using a trans-well chamber. When hBMECs were immunostained for tight junctional markers ZO-1, VE-cadherin, claudin-5, and Occludin, an increased endothelial cell density in co-cultures with iMSCs compared to hBMEC monocultures, with junctional markers detectable in both conditions. (Figure 2A). The trans-endothelial electrical resistance (TEER) was also measured in hBMECs daily over the course of 7 days (Figure 2B). Co-culture with MSCs have been shown to increase TEER and decrease permeability in endothelial cells (Tian et al., 2017). Consistently, the presence of iMSCs significantly increased TEER values in hBMECs compared with hBMECs cultured alone (Figure 2C). Particularly, comparison of TEER values on day 7 shows doubling resistance in hBMECs when in contact with *APOE3Ch* iMSCs. Contact with *APOE3* iMSCs also significantly increased TEER in hBMECs, albeit modestly. Overall, co-culture with *APOE3Ch* iMSCs yielded significantly greater TEER in hBMECs compared with *APOE3* iMSCs (Figure 2D).

### 3.3 *APOE3Ch* iMSCs support endothelial tube formation

**FIGURE 3:**
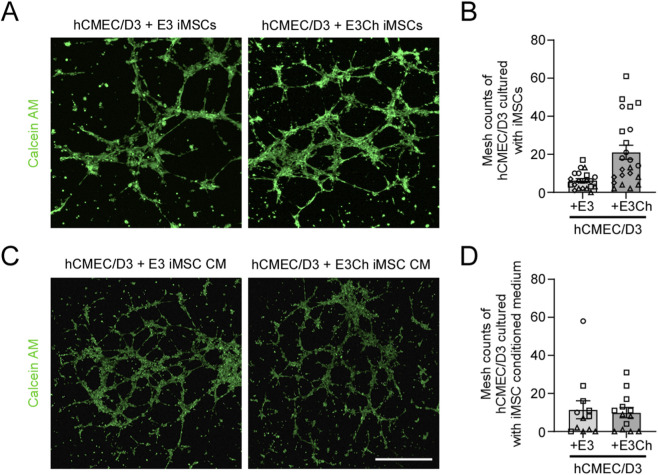
*APOE3Ch* iMSCs facilitate endothelial tube formation. **(A,C)** Representative images of hCMEC/D3 endothelial tube formation **(A)** when co-cultured with *APOE3* or *APOE3Ch* iMSCs, **(C)** or their respective conditioned media. Scale bar = 100 µm. **(B,D)** Quantification of hCMEC/D3 mesh formation **(B)** co-culture with *APOE3* or *APOE3Ch* iMSCs or **(D)** their respective conditioned media. Data shown as mean ± SEM (n = 4 independent experiment for co-culture and n = 3 independent experiments for conditioned media, technical replicates from each experiment are represented by a distinct symbol, for display only). Paired t-test performed on n = 3 to 4 independent experiments.

MSCs can function as perivascular-like support cells, physically associating with endothelial networks and promoting their architecture through direct contact mechanisms (Au et al., 2008; Crisan et al., 2008). We confirmed this phenotype in hCMEC/D3 cells (Supplementary Figure S2A). Tube formation was significantly increased when culturing these endothelial cells with serum-free iMSC media even in the absence of iMSC, although iMSC significantly improved this outcome (Supplementary Figure S2B). After validation of iMSCs perivascular support, we evaluated the effects of *APOECh* on endothelial angiogenic behavior by assessing tube formation in endothelial cells exposed to *APOE3* or *APOE3Ch* iMSCs (Figure 3A). Tube formation in hCMEC/D3 was visualized using Calcein AM live staining. Confocal images were analyzed for total mesh count as a quantitative readout of network complexity. hCMEC/D3 cells co-cultured with *APOE3Ch* iMSCs displayed more tubing than those co-cultured with *APOE3* iMSCs, with a mesh count tripled with *APOE3Ch* although this was not significant (Figure 3B). However, exposures of endothelial cells to the secretome from *APOE3Ch* and *APOE3* iMSCs did not lead to any difference in the tube formation (Figures 3C,D). These results indicate that direct contact of hCMEC/D3 with *APOE3Ch* iMSCs is necessary to enhance the complexity of the formed vascular network compared with *APOE3*.

### 3.4 Secretome from *APOE3Ch* iMSCs facilitate neurite outgrowth

**FIGURE 4:**
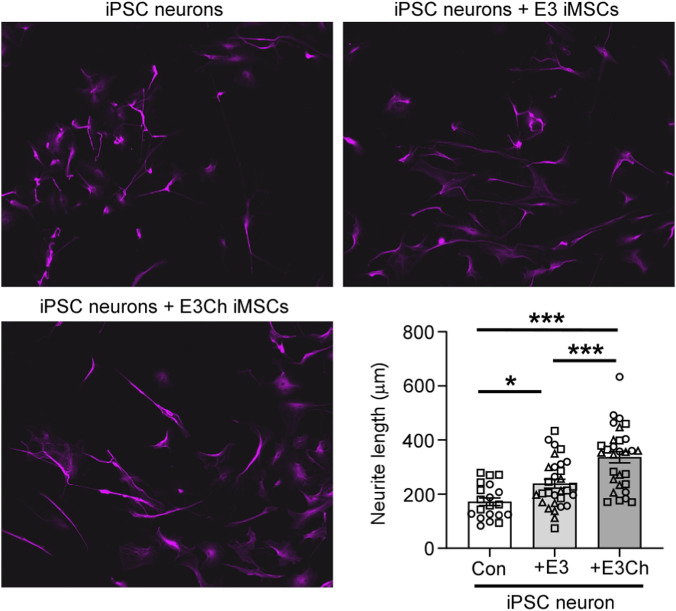
*APOE3Ch* iMSCs promote neurite extension in iPSC-derived neurons. Representative images of iPSC-derived neurite growth in the presence of conditioned media from *APOE3* iMSCs, *APOE3Ch* iMSCs or control media (BrainPhys) stained with TUJ1 (magenta). Quantification of individual neurite length measured using ImageJ/Fiji plug-in Simple Neurite Tracer (SNT). Data represented as mean ± SEM (n = 3 independent experiments, technical replicates from each experiment are represented by a distinct symbol, for display only). *p < 0.05, ***p < 0.001 by mixed-effects model on n = 3 independent experiments with the Geisser-Greenhouse correction to account for matched values, and Tukey’s *post hoc* multiple comparisons test.

Having observed that *APOE3Ch* iMSCs improved endothelial barrier function and angiogenesis property, we next assessed how their secretome influence neurite extension of iPSC-derived neurons (Figure 4). The iPSC neurons were cultured under normal conditions for a week, after which neuron culture media was substituted for *APOE3* or *APOE3Ch* iMSC conditioned media for an additional 2 weeks prior to staining and imaging. Individual neurite lengths were quantified as a measure of neurite outgrowth. While the secretome of *APOE3* iMSCs modestly but significantly increased the length of neurites in iPSC-derived neurons compared to those cultured in control media, the secretome of *APOE3Ch* iMSCs induced a superior effect on the neurite length (Figure 4).

### 3.5 *APOE3Ch* iMSCs display altered mitochondrial stress responses

**FIGURE 5:**
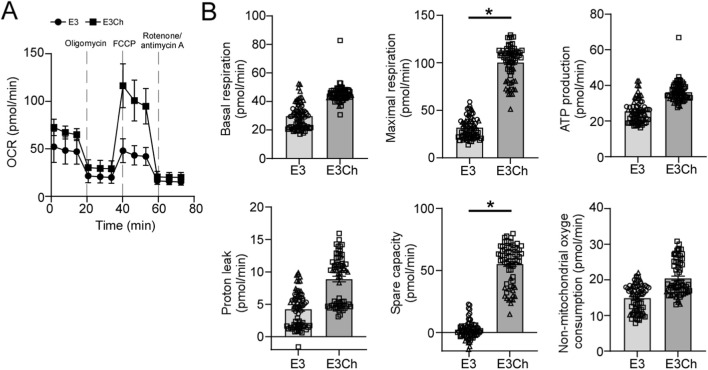
*APOE3Ch* iMSCs display higher mitochondrial respiration rate. **(A)** Oxygen consumption rate (OCR) of *APOE3* iMSCs or *APOE3Ch* iMSCs was measured under basal conditions and was recorded following sequential injections of oligomycin, carbonyl cyanide-p-trifluoromethoxyphenylhydrazone (FCCP), and rotenone/antimycin A at the indicated time points. OCR profiles normalized to cell numbers. **(B)** Quantification of basal respiration, maximal respiration, ATP production, proton leak, spare respiratory capacity, and non-mitochondrial oxygen consumption. Data shown as mean ± SEM (n = 3 independent experiments, technical replicates from each experiment are represented by a distinct symbol, for display only). *p < 0.05 by two-tailed paired Student’s t-test.

Building on the observed effects of *APOE3Ch* iMSCs on endothelial cells and neurons, we examined whether these increased trophic functions were associated with altered iMSC mitochondrial metabolism by performing a Seahorse XF Mito Stress Test. OCR was measured over the course of the test, following sequential injections of drugs that selectively shut down different components of the electron transport chain. The OCR traces throughout the mitochondrial stress assay showed distinct responses in *APOE3* and *APOE3Ch* iMSCs (Figure 5A). Key parameters derived from OCR include basal respiration, maximal respiration, ATP production, proton leak, spare respiratory capacity and non-mitochondrial oxygen consumption. Particularly, maximal respiration and spare respiratory capacity were significantly higher in *APOE3Ch* iMSCs than *APOE3* iMSCs (Figure 5B).

## 4 Discussion

The discovery of the protective effects of *APOE3Ch* against AD (Arboleda-Velasquez et al., 2019) paved the way for mechanistic studies aimed at understanding protective APOE functions. Several groups have confirmed the protective effects of *APOE3Ch* both *in vivo* and *in vitro*. For instance, *APOE3Ch* expression leads to reduced levels of NFTs and Aβ plaques in an amyloid model crossed with *APOE3Ch* knockin mice inoculated with human tau (Chen et al., 2024). The Christchurch mutation also resulted in decreased tauopathy in an APOE4 background (Nelson et al., 2023), mouse Apoe background (Tran et al., 2025), and in AAV-mediated expression models (Gunaydin et al., 2024). *APOE3Ch* was also reported to modulate immune-related pathways in tauopathy mice (Naguib et al., 2025) and in iPSC-derive microglia like cells (Sun et al., 2024). Since most studies have focused on amyloid and tau pathologies, the influence of *APOE3Ch* on neurovascular processes remains poorly understood. Simultaneously, MSCs have emerged as a promising platform for neurovascular repair due to their perivascular origin and paracrine capacity to modulate endothelial, metabolic, and neuronal function (Davis et al., 2021). This is particularly relevant to both aging and neurodegenerative diseases which are associated with BBB impairment and NVU dysfunction (Vargas-Rodriguez et al., 2023). As such, these two areas are integrated in our study by using iMSCs to examine whether *APOE3Ch*, compared with *APOE3*, would enhance neurovascular-relevant trophic properties of MSCs, including immunomodulation, barrier integrity, angiogenesis, mitochondrial metabolism and neuronal support.

We confirmed that these iMSCs secrete APOE. While there were no significant differences in intracellular APOE levels, they were increased in the conditioned medium from *APOE3Ch* iMSCs compared to APOE3 iMSCs. *APOE* mRNA levels were not significantly different between the two iMSC lines. These differences in secreted APOE levels despite comparable transcription point to either structural changes or potential variations in protein processing and clearance. The latter would be consistent with differences in APOE protein binding, particularly to HSPGs (Arboleda-Velasquez et al., 2019; Guo et al., 2025). This is especially relevant to MSC biology given their expression of HSPG core proteins that modulate their function (Okolicsanyi et al., 2018; Saez et al., 2014). Both iMSC lines successfully suppressed inflammatory activation in LPS-treated THP1 monocytes with significantly reduced activation of the NFκB pathway, attesting to their immunomodulatory effects (Ko and Oh, 2023). Although the differences were very modest, we found that *APOE3Ch* iMSCs have stronger immunomodulatory properties than *APOE3* iMSCs. Interestingly, immune competence is one of the MSC core paracrine functions in the CNS (Li et al., 2022). Indeed, a recent report showed that *APOE3Ch* reduces immune activation in microglia in a mouse model of tauopathy compared with *APOE3* (Naguib et al., 2025). One limitation of our study is that immune modulatory effects of *APOE3* and *APOE3Ch* iMSCs were not assessed in cell types that are relevant to neurodegeneration, such as endothelial cells, microglia, or neurons. Given the effects we observe on activated THP1 cells, and reports of decreased neuroinflammation in several *APOE3Ch* mouse models of AD, we suspect that increased immune competency of *APOE3Ch* iMSC would be observed in other activated cell models, but additional studies are needed to address this.

BBB integrity and angiogenic remodeling are critical to NVU maintenance and repair, particularly in the context of aging and disease. We observed that *APOE3Ch* iMSCs increase TEER and facilitate tube formation of endothelia cells more effectively than *APOE3* iMSCs. Pericytes isolated from APOE3-targeted replacement (TR) mice support greater endothelial barrier integrity and tube-like structure formation than APOE4 pericytes (Yamazaki et al., 2020). Pericytes are cells from a mesenchymal lineage that surround the outside of blood vessels. They are in direct contact with BECs on the luminal side and with neurons and glial cells in the brain (Attwell et al., 2016). Their role is to structurally support, promote angiogenesis and regulate the permeability of vasculature in the brain (Procter et al., 2021). As MSCs share many functional properties with pericytes, our results suggest that *APOE3Ch* may influence cerebrovascular support functions mediated by MSCs and pericytes. MSCs have also been shown to secrete pro-angiogenic factors such as IL-6, VEGF, and MCP-1 that promote endothelial network formation as secretome from MSCs stimulate endothelial tube formation *in vitro* (Hung et al., 2007). In our system, differences between APOE3Ch and APOE3 in the endothelial tube formation were observed only under conditions involving direct iMSC-endothelial contact. Thus, *APOE3Ch* may influence specific signaling pathways or cell-to-cell transfer between iMSCs and endothelial cells through direct cell contact, although further studies are necessary. Beyond structural stabilization of the BBB, enhanced endothelial integrity may limit paracellular permeability to inflammatory mediators, preserve regulated transport processes across the barrier, and contribute to a more stable neurovascular microenvironment, particularly in the context of AD-associated vascular dysfunction (Yue et al., 2024).

In addition, preservation or restoration of neuronal connectivity is of increasing interest in the context of aging-associated neurovascular dysfunction in neurodegeneration. MSCs are an attractive cell-based therapeutic approach due to their ability to secrete neurotrophic factors that support neuronal survival and neurite extension (Seo and Cho, 2012; Uccelli et al., 2008). Indeed, iMSC conditioned media has been shown to modulate neurite outgrowth of iPSC-derived neurons (Kawatani et al., 2025). APOE plays a central role in neuronal lipid homeostasis, membrane remodeling, and neurite dynamics in an isoform dependent manner, with protective variants supporting greater structural resilience and synaptic integrity (Huang and Mahley, 2014; Lindner et al., 2024). APOE has been shown to directly regulate neurite outgrowth in an isoform-dependent manner, with APOE4 inhibiting neurite extension in multiple systems (Hussain et al., 2013; Nathan et al., 2002; Zhao et al., 2017). When the effects of *APOE3* and *APOE3Ch* iMSC secretome on iPSC-derived neurons neurite extension, we found that secretome of *APOE3Ch* iMSCs resulted in significantly longer neurites than that of *APOE3*. Although our study did not include *in vivo* experiments, emerging work on *APOE3Ch* knock-in mouse models suggest that this protective variant can influence neuron-relevant outcomes. In the context of AD mouse models, *APOE3Ch* has been associated with reduced tau propagation, decreased synaptic loss, and improved neuronal network function compared to APOE3 wild-type controls. While these effects are often mediated through altered glial responses, the downstream consequences directly impact neuronal integrity (Chen et al., 2024; Nelson et al., 2023; Tran et al., 2025). *APOE3Ch* iMSC promoting enhanced neurite extension is therefore consistent with the broader literature relating *APOE3Ch* to neuronal resilience phenotypes. Overall, our findings align with a therapeutic model in which *APOE3Ch* iMSCs provide paracrine neuronal support that complements their beneficial effects on vascular integrity and angiogenic behavior.

We observed several features consistent with enhanced neurovascular support in *APOE3Ch* iMSCs compared with *APOE3*. Since these phenotypes depend on energy-depleting processes, we investigated whether these functional effects might be supported by differences in iMSC mitochondrial metabolism, which can impact their secretory and interactive properties. Our results found higher maximal respiration and increased spare respiratory capacity in *APOE3Ch* iMSCs relative to *APOE3*, which can be interpreted as an expansion of mitochondrial oxidative capacity and an increased bioenergetic reserve. *APOE3Ch* iMSCs showed a consistent directionality toward higher basal respiration, ATP-linked respiration, proton leak, and non-mitochondrial oxygen consumption across independent experiments, although these did not reach statistical significance. This pattern is in line with increased respiratory activity and potentially altered coupling. However, given the variability across independent experiments and the limited biological replication, these findings should be interpreted cautiously and confirmed in future studies with increased sample size. Nevertheless, the ability of *APOE3Ch* iMSC to meet elevated energetic demand or adapt to metabolic stress as depicted by significantly increased maximal respiration and spare respiratory capacity relative to *APOE3* may enable greater paracrine potential. This is consistent with reports that MSC mitochondrial metabolism is linked to secretion of trophic factors and intercellular communication, in turn impacting function in other cells (Yan et al., 2021; Wang et al., 2025). Collectively, these metabolic enhancements may underpin the improved neurovascular supportive capacity of iMSCs, highlighting a potential link between APOE3Ch and bioenergetic fitness.

In summary, our study demonstrates that iMSCs expressing the protective *APOE3Ch* variant exhibit enhanced metabolic fitness, strengthen endothelial barrier integrity, promote angiogenesis, and support neuronal outgrowth compared with *APOE3* iMSCs. These findings highlight a multifaceted mechanism in which MSC bioenergetics and APOE converge to modulate NVU function and neurotrophic support. By linking iMSC metabolic state, *APOE* variants, and functional outcomes in endothelial and neuronal cells, our results provide a rationale for considering *APOE3Ch* iMSCs as promising therapeutic strategy to mitigate NVU vulnerability in aging and neurodegeneration. Further studies investigating *in vivo* efficacy and the molecular mediators of these effects will be critical for translating these findings into clinically relevant interventions.

## Data Availability

The raw data supporting the conclusions of this article will be made available by the authors, without undue reservation.
