# Supplementary material for: APOE3-Christchurch variant enhances neurovascular support functions of iPSC-derived mesenchymal stromal cells
Source: Front Mol Biosci. 2026 Jun 1;13:1778856. doi: 10.3389/fmolb.2026.1778856 (PMC13265337; doi:10.3389/fmolb.2026.1778856)
Supplement: Supplementary file 1 [file DataSheet1.pdf]

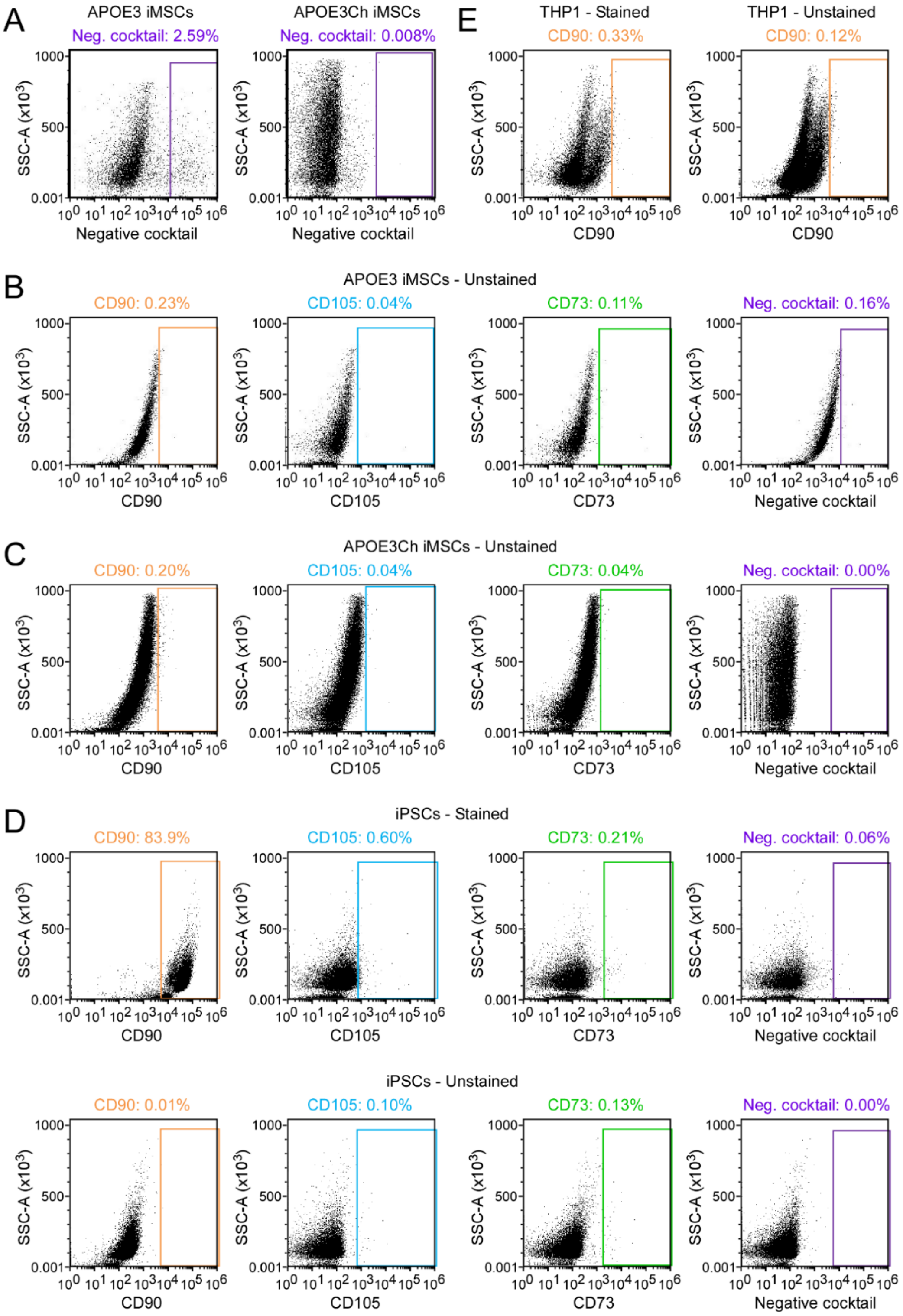

Supplemental Figure 1

(A) Successful gating of *APOE3* and *APOE3Ch* iMSCs FACS with negative antibody cocktail present in <5% of the cell population. (B, C) FACS analysis of unstained APOE3 (B) and APOE3Ch (C) iMSCs shows the absence of positivity for CD90, CD105, and CD73, as well as successful gating. (D) iPSCs were used as a negative control for CD105 and CD73 as iPSCs do not express these markers, however they are positive for CD90 consistent with the literature. (E) THP1 cells were used as a negative control for CD90.

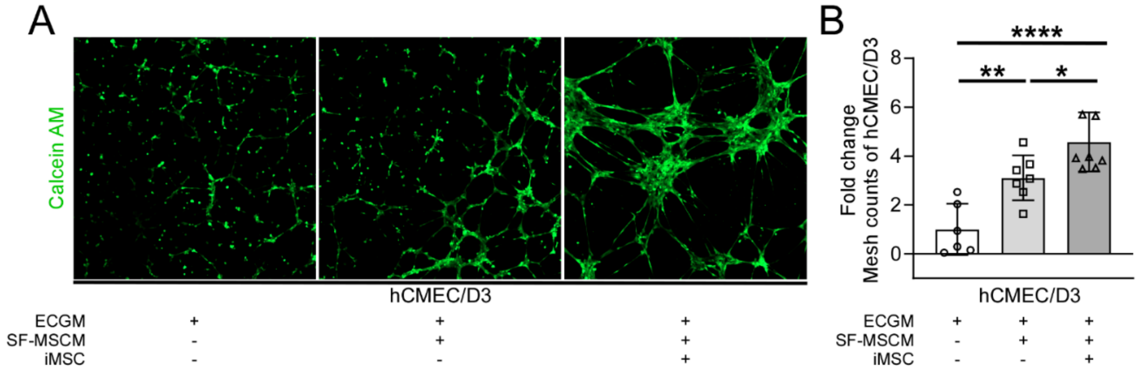

Supplemental Figure 2

(A) Representative images of hCMEC/D3 endothelial tube formation in EGCM media only (left), and in 1:1 EGCM:SF-MSCM media in the absence (middle) or presence of iMSC (right) (-iMSC and +iMSC respectively. (B) Quantification of hCMEC/D3 mesh formation after culture with SF-MSCM and the inclusion of iMSC, Data shown as mean ± SD (n=6-8 technical replicates). Ordinary one-way ANOVA, with Tukey's post hoc multiple comparisons test. ECGM: Endothelial cell growth media. SF-MSCM: Serum-free MSC media.

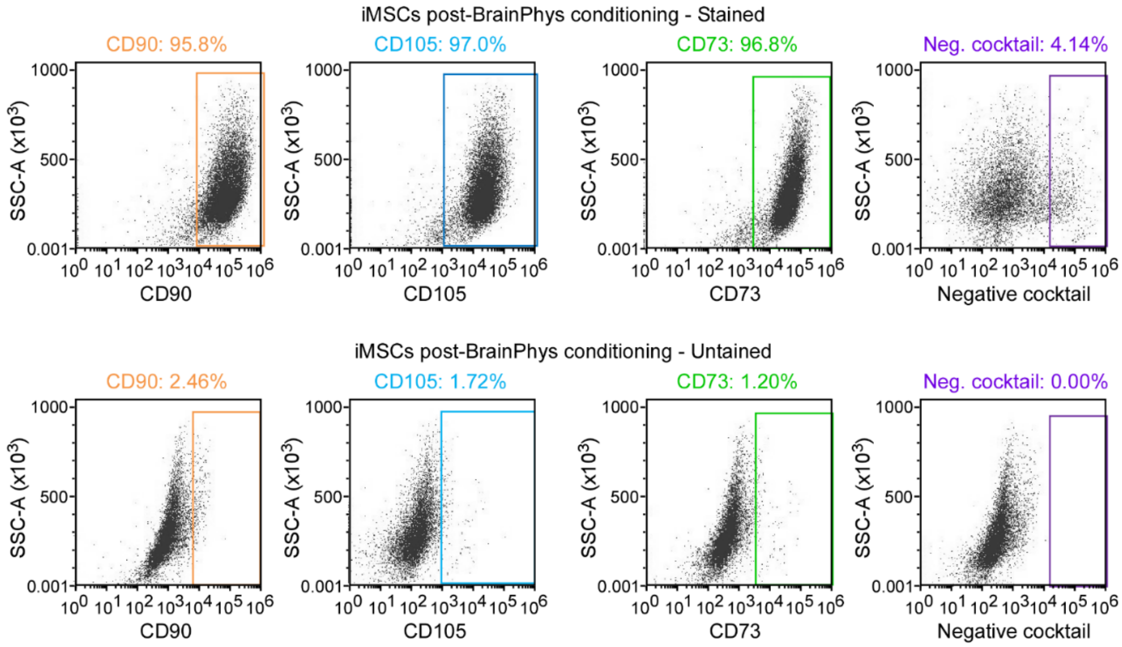

Supplemental Figure 3
(Top) FACS analysis confirming that MSC identity is retained in iMSCs following conditioning with BrainPhys, with CD90, CD105 and CD73 expressed in >95% of the cell population (Bottom) Unstained cells were used as negative control.
