# Supplementary material for: APOE3-Christchurch variant enhances neurovascular support functions of iPSC-derived mesenchymal stromal cells
Source: Front Mol Biosci. 2026 Jun 1;13:1778856. doi: 10.3389/fmolb.2026.1778856 (PMC13265337; doi:10.3389/fmolb.2026.1778856)
Supplement: Supplementary file 2 [file DataSheet2.zip › WB APOE/APOE WB_Layout.pdf]

LAYOUT:

LD|1|2|3|4|5|6|7|8|9|10|11|12|13|14|15|16|17|18|19|20|21|22|23|24|

SAMPLES

LD = Ladder; Lys = Lysate; CM = Conditioned media

1: Lys 1A_1	9: Lys 2A_1	17: Lys 3A_1
2: Lys 1A_2	10: Lys 2A_2	18: Lys 3A_2
3: Lys 1B_1	11: Lys 2B_1	19: Lys 3B_1
4: Lys 1B_2	12: Lys 2B_2	20: Lys 3B_2
5: CM 1A_1	13: CM 2A_1	21: CM 3A_1
6: CM 1A_2	14: CM 2A_2	22: CM 3A_2
7: CM 1B_1	15: CM 2B_1	23: CM 3B_1
8: CM 1B_2	16: CM 2B_2	24: CM 3B_2

3 independent differentiations (color coded)

2 replicates per measurements

A = *APOE3*, B = *APOE3Ch*
