# Supplementary figures and images for: APOE3-Christchurch variant enhances neurovascular support functions of iPSC-derived mesenchymal stromal cells
Source: Front Mol Biosci. 2026 Jun 1;13:1778856. doi: 10.3389/fmolb.2026.1778856 (PMC13265337; doi:10.3389/fmolb.2026.1778856)

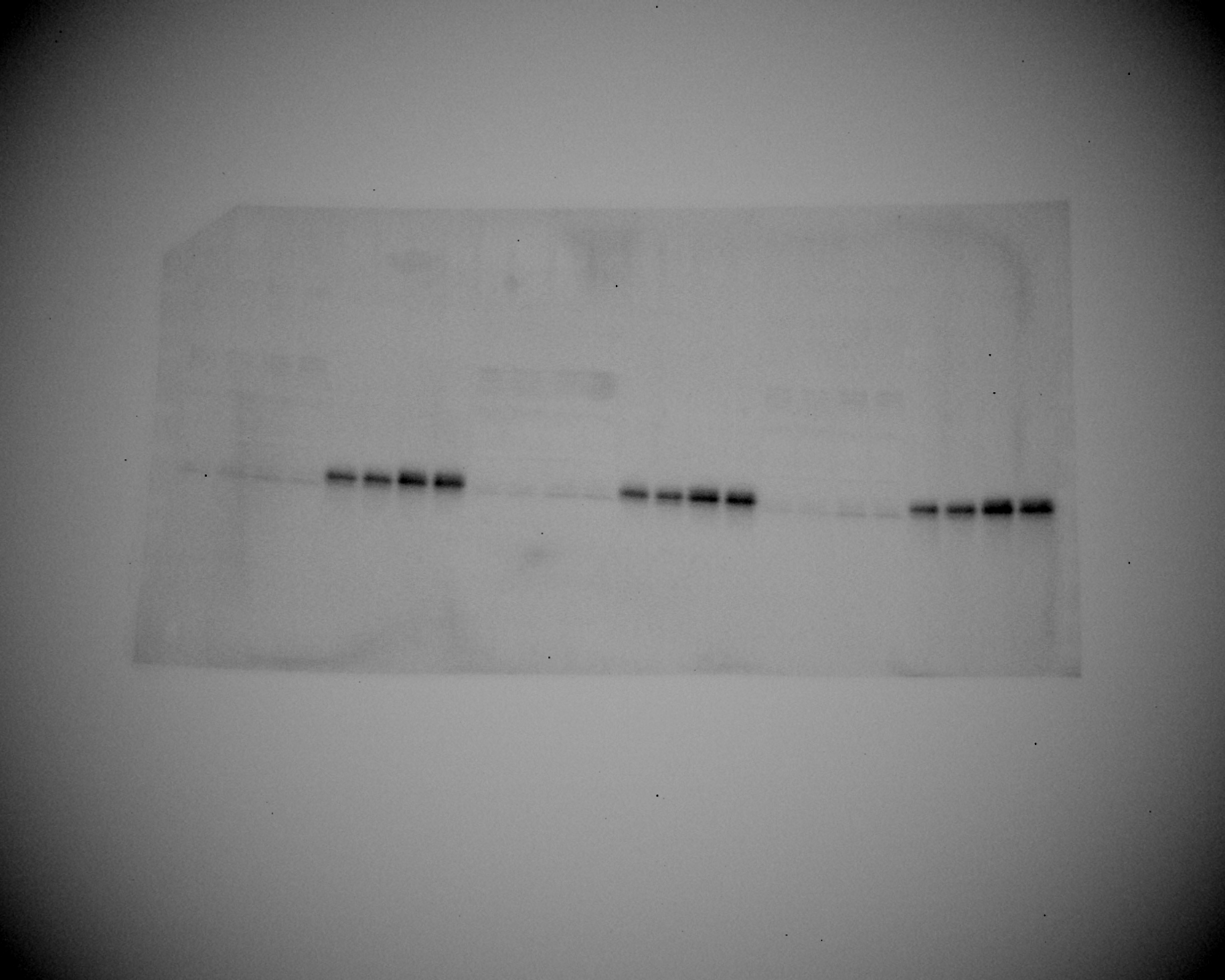

Supplement: Supplementary file 2 [file DataSheet2.zip › WB APOE/APOE_WB_3.tif]
